# Implementation of an educational module on nosocomial infection control measures: a randomised hospital-based trial

**DOI:** 10.1186/s12912-021-00551-0

**Published:** 2021-02-17

**Authors:** Gamil Ghaleb Alrubaiee, Anisah Baharom, Ibrahim Faisal, Hayati Kadir Shahar, Shaffe Mohd Daud, Huda Omer Basaleem

**Affiliations:** 1 Department of Applied Medical Sciences, Faculty of Medical Sciences, Al-Razi University, Sana’a, Yemen; 2 Department of Community Health, Faculty of Medicine and Health Sciences, Universiti Putra, Seri Kembangan, Malaysia; 3 Department of Foundations of Education, Faculty of Educational Studies, Universiti Putra, Seri Kembangan, Malaysia; 4 Department of Community Medicine and Public Health, Faculty of Medicine and Health Sciences, University of Aden, Aden, Yemen

**Keywords:** Nosocomial infections, Cross-infection, Educational module, Infection control, Nurses, Yemen

## Abstract

**Background:**

Previous cross-sectional studies have reported limited knowledge and practices among nurses regarding controlling nosocomial infections (NIs). Even though health institutions offer many irregular in-service training courses to solve such issues, a three year-nursing educational programme at institutions is not adequate to enable nurses to handle NIs. Therefore, this study aims to evaluate the implementation of an educational module on NIs control measures among Yemeni nurses.

**Methods:**

A single-blinded randomised hospital-based trial was undertaken involving 540 nurses assigned to two intervention groups and a waitlist group. Intervention group-1 received a face-to-face training course comprising 20 h spread over six weeks and a hard copy of the module, while intervention group-2 only received the hard copy of the module “without training”. In contrast, the waitlist group did not receive anything during the period of collecting data. A self-administered NI control measures-evaluation questionnaire was utilised in collecting the data from the participants; before the intervention, at six weeks and 3 months after the end of the intervention. The period of data collection was between 1^st^ May and 30^th^ October 2016.

**Results:**

The results from collecting and analysing the data showed a statistically significant difference in the mean knowledge scores between the intervention groups that were detectable immediately post-intervention with a mean difference (MD) of 4.31 (*P* < 0.001) and 3 months after the end of the intervention (MD = 4.48, *P* < 0.001) as compared to the waitlist group. Similarly, the results showed a statistically significant difference in the mean practice scores between the intervention groups immediately post-intervention (MD = 2.74, *P* < 0.001) and 3 months after the intervention (MD = 2.46, *P* < 0.001) as compared to the waitlist group. Intervention-1 (face-to-face training + module) was more effective than intervention-2 (module only) in improving Yemeni nurses’ knowledge and practices regarding NI control measures compared to the waitlist group.

**Conclusion:**

The findings of this study found that intervention-1 could be offered to nurses in the form of an in-service training course every six months. The NI course should also be included in nursing curricula, particularly for the three-year-nursing diploma in Yemen.

**Trial registration:**

Nosocomial infection educational module for nurses ISRCTN19992640, 20/6/2017. The study protocol was retrospectively registered.

**Supplementary Information:**

The online version contains supplementary material available at 10.1186/s12912-021-00551-0.

## Background

Nosocomial infections (NIs) are defined as an infection acquired following 48 h of patient admission to a given hospital or other health care institutions resulting from delivering healthcare services to patients [1]. As such, NIs represent a considerable safety concern for both providers and consumers of healthcare services. NIs also arise due to morbidity and mortality. It is estimated that almost 1.4 million people worldwide are infected each year, with a prevalence rate ranging between 3.0 and 20.7% [2]. In contrast, the estimated prevalence rate in high-income countries is reported to be between 3.5 and 12%, and between 5.7 and 19.1% in middle-income countries [3, 4]. Thus, such infections have a serious impact on the quality of patient health care outcomes [5], and increased morbidity, leading to unnecessary deaths and additional cost [6].

Nurses are primarily responsible for implementing daily patient care activities in hospitals and other health institutions that involve more contact with patients than other healthcare workers (HCWs). Consequently, nurses are more exposed to various NIs and play a vital role in transmitting NIs. As such, the compliance of nursing staff with infection control measures is necessary for preventing and controlling NIs [7]. However, the compliance of nurses with infection control measures requires an appropriate level of knowledge to enable them to apply appropriate infection control measures in the clinical setting [8].

Despite the effectiveness of many previous educational programmes developed to improve the level of knowledge and practices of nurses in applying infection control measures reported in earlier research, in practice, nurses’ knowledge and practices of NIs are inadequate [9]. This is evidenced in Yemen, where nurses display insufficient knowledge, applying a range of various infection control measures, suggesting the need to enhance the knowledge and practices of Yemeni nurses’ [10]. According to Sherah [11], only 3.4% of Yemeni nurses displayed an adequate level of practice regarding infection control measures.

Based on the three-year nursing curriculum in Yemen, nurses have limited exposure to topics and knowledge about infection control measures during their term of study in the three-year diploma programme [12]. Furthermore, the study programme is not an integrated education unit but is delivered within many different subjects. Importantly, there is no clear evidence to suggest that the three-year nursing diploma programme had any formal or specific unit on infection control measures integrated into one course, along with a standard outline and specific objectives to ensure the effective delivery of such topics would lead to attaining the intended outcomes [13]. Another aspect of the problem is that Yemeni nurses are not subjected to in-service training on infection control measures while engaged in their professional work at public hospitals [14].

Most of the previous studies suggest that developing and implementing an educational programme targeting infection control precautions for healthcare providers on a regular basis should ensure their compliance with infection control policies, practices and guidelines [15]. However, providing an educational intervention, covering all NI control measures, is not easy since it requires dealing with multidimensional themes and issues. In addition, most of the studies reviewed in this field in the context of educational interventions focused on a single infection control measure, such as hand hygiene, medical-surgical asepsis, sterilisation and disinfection, isolation precautions.

The only exception was found in two studies by Taha [16], and Galal et al. [17] that addressed the standard precaution components and a third study by Wu et al. [18] that addressed both standard precautions and additional precaution components. Of these studies, one study employed a pre- and post-test, non-random sampling, without a control group, while another study used the same design but with random sampling, and the third study employed a quasi-experimental design. However, none of these programmes were evaluated in a randomised control trial (RCT) design.

Furthermore, the reviewed studies used either a one group pretest-posttest design or using two groups to evaluate the educational intervention’s effectiveness. The only exception was the study by Ghezeljeh et al. [19] that used three groups. Using such a design, the researcher compares two intervention groups with a control group, which allows a fair comparison between two intervention groups to the same control group, much faster and more efficiently [20], less administrative burdens and costs [21] and under a single protocol at one time. Hence, it utilises the same advantages, such as inclusion/exclusion criteria [22]. Thus, evaluating the effectiveness of educational interventions using a different design and in different contexts is required to gain further evidence on its effectiveness.

Accordingly, an educational intervention of NI infection control measures addressing the different aspects is required. This can be achieved by designing the intervention in an integrated and needs-based educational module, through multimodal teaching strategies and employing a three-group RCT design in evaluating this approach. In the present study, two intervention groups are compared to a waitlist group. Intervention group-1 received (face-to-face training + module), intervention group-2 received (module only “without training”), and the waitlist group did not receive anything during data collection. The purpose of using intervention group-2 was to evaluate whether the intervention as a self-study module “without training” would be effective in improving nurses’ knowledge and practice regarding NI control measures. Therefore, the current study aimed to evaluate the effectiveness of an educational module on NI control measures to improve the level of knowledge and practice among Yemeni nurses.

## Method

### Study design and setting

**Fig. 1:**
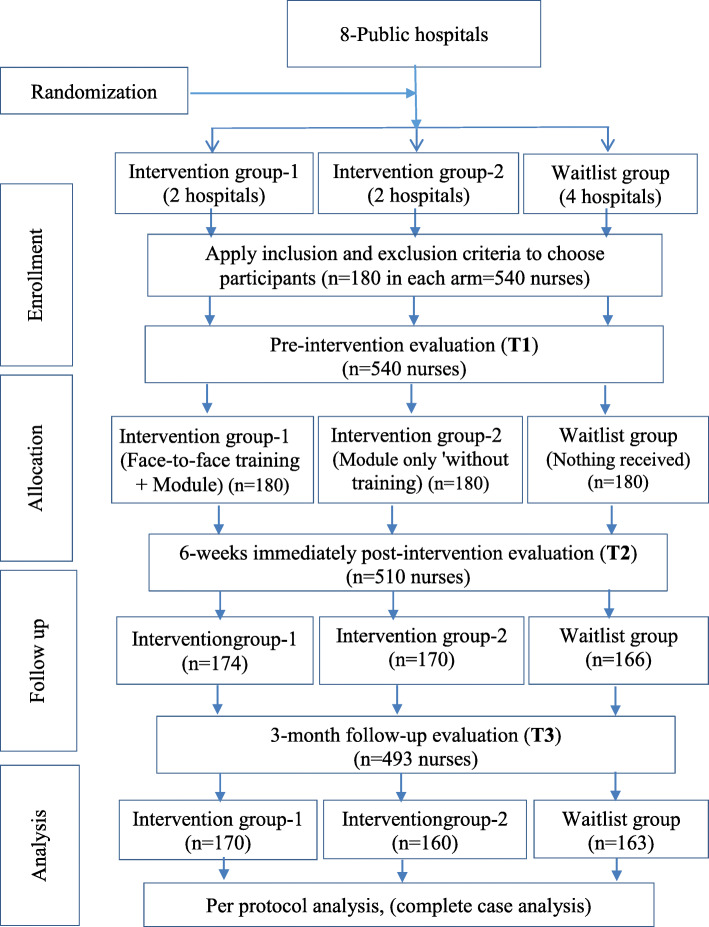
Flow-chart of the study design and outcome evaluation

A single-blinded randomised hospital-based trial was designed and conducted in eight (8) public hospitals in the Aza’al Region in the Republic of Yemen. This approach was adopted in which the hospitals and participants were unaware of the random allocation of the educational method until the intervention had begun. After that, blinding was not possible to maintain given the hospitals had received the intervention. The overall flow-chart of the study is shown in Fig. 1.

### Sample and sampling method

Yemeni nurses of both genders employed in public hospitals in Yemen, having a three-year nursing diploma following secondary school, and having a minimum of one-year working experience were eligible to participate in this study. However, since the study aimed to evaluate the effectiveness of the module among Yemeni nurses, foreign nurses were excluded. Two-stage cluster sampling was utilised. In the first stage, simple random sampling was utilised to select three cities from five cities located in the Aza’al Region. The eight (8) public hospitals in the three selected cities were allocated randomly as the intervention and waitlist groups. In the second stage, of the eight (8) hospitals, from a total of 1262 nurses, 540 nurses were randomly selected who provided direct care to patients (180 nurses in each group), using proportional to size sample design. The nurses’ names were obtained from the hospitals, which were used to calculate the required sample size from each hospital based on the ratio of nurses working in each hospital to the total number of nurses working in all selected hospitals (Additional file 1).

### Sample size

The sample size was estimated based on two-sample group proportions [23]. It was found that a sample size of 93 nurses (in each group) would be required to obtain 80% power to detect a difference of 0.20 between the groups, considering that both knowledge and practice in the previous study were P1 = 0.70; and P2 = 0.50, respectively [24]. Therefore, P was = (P1 + P2)/2 = 0.6.

Also, given the participating nurses were nested within the eight (8) hospitals, the design effect was considered. The participants per hospital were assumed to be 16, in which the intra-cluster coefficient (ICC) was 0.05. This resulted into a design effect =1+ (m-1) × ICC =1+ (16–1) × 0.05 = 1.75. Next, by multiplying this result by the required sample size, *N* = 93, the sample size became 93 × 1.75 = 163. Assuming an attrition of 10% during the study, n was = 163 + 10% of 163 = 163 + 17 = 180. Thus, the total sample size in each arm was *n* = 180, where Z1-α/2 and Z1-β were assumed to be 1.96 and 0.84, respectively.

### Randomisation and the blinding process

A simple randomisation technique was adopted to allocate the eight (8) selected hospitals to the three groups (two intervention groups, and the waitlist group). The hospitals were randomly coded, signified as A, B, C, D, E, F, G, and H. The name of each hospital was written on eight small (8) pieces of paper and placed inside a box. The hospitals’ names were then drawn from the box independently applying the lottery method. The name of the first drawing was assigned to hospital A, second drawing to hospital B, and so on until hospital H. These allocation codes were masked from the investigator who then allocated each hospital to one of the three waitlist/intervention groups. The study groups’ names were then sealed in opaque envelopes that were sequentially numbered and individually sealed to avoid any selection bias. An independent statistician using a random number generator programme allocated the hospitals to one of the study groups with a ratio of 2:2:4; intervention group-1 having two (2) hospitals, intervention group-2 having two (2) hospitals and the waitlist group having four (4) hospitals. Notably, the group allocation was not released to the respondents before obtaining their written consent, and the baseline data had been collected.

### Educational intervention

The educational module aimed to enhance the nurses’ knowledge about NI control measures and enable them to apply these measures in real clinical situations. Three-intervention groups were designed for this very purpose. Intervention group-1 received a face-to-face training course consisting of three educational units, 20 h in total, spanning over six weeks. They also received the learning materials; a hard copy of the educational module (60-pages) and a CD containing short-videos related to NI control measures. Intervention group-2 only received the hard copy of the educational module and the CD “without training”. In contrast, the waitlist group did not receive any material or training during data collection but were given the educational materials following completion of the study as an ethical consideration.

Delivery of the multimodal learning strategies was utilised for intervention group-1 in ten (10) successive training sessions. In order to ensure the successful implementation of the sessions, a comprehensive communication strategy was adopted before its implementation. The infection control practitioner (ICP) in the selected hospitals was appointed as a contact person, responsible for coordinating all activities and contacts between the hospital and researchers. In coordinating with the contact person(s), the hospital’s authority and participants, a flexible schedule was provided, and the participants were then divided into 12 small groups, each involving 15 participants. This was followed by allowing the participants to attend the face-to-face training course either in the morning or during their evening shifts. This helped address and overcome any issues regarding the participants’ non-attendance expected during the training sessions and refusal from the hospital’s administration area to conduct the training. The training course was delivered by three (3) specialised nurses who held a master’s degree in nursing and with previous working experience in infection control. A guest from the Central Sterile Supply Department (CSSD) also assisted in one training session related to sterilisation procedures. A summary of the educational module is provided in Table 1.

**Table 1:** Summary of the Educational Module (Themes, Content, and Teaching Strategies)

Theme	Content	Teaching strategies
**Unit one:** Introduction	Overview on NIs. Point of care risk assessment.	Interactive lecture (slides), brainstorming and small group discussion
**Unit Two:** Prevention of person-to-person transmission	Hand hygiene (HH). Personal protective equipment (PPE). Safe injection practices.	Interactive lecture with slides, small group discussion, group work assessment, short video demonstration followed by discussion, hands-on practice.
**Unit Three:** Prevention of transmission from the hospital environment	Reprocessing of patient care equipment. Routine hospital cleaning. Safe linen handling. Safe hospital waste handling and disposal. Source control.	Interactive lecture with slides, small group discussion, video demonstration, role play, performance-based assessment
**Summary and evaluation**	Module reflection	Module evaluation and open discussion.

### Study instrument

A self-administered questionnaire was developed and used to evaluate the outcomes from the study at three stages [25]. The questionnaire comprised of 45-items to collect and assess the knowledge and practice of NI control measures of the nurses. The questionnaire divided into three sections. The first section covered the socio-demographic data [profile] of the participants, the second section addressed the knowledge of nurses, and the third section covered the practices of nurses regarding NI control measures. Questionnaire items were closed-ended questions. The nurses’ knowledge was assessed using 30-items with “Correct”, “Incorrect” and “I don’t know” options, while the nurses’ practice was assessed presenting two real-world scenarios related to NI control measures followed by 15-items with “Yes”, and “No” and “I don’t know” options. Each “incorrect” or “I don’t know” response was given a 0 score, and each correct response was given a score of 1. Based on this scoring system, the maximum and minimum scores for the correct knowledge responses ranged between 1 to 30, while the correct practice responses scored between 1 and 15.

### Validity and reliability

The instrument’s construct validity was assessed based on a pilot study that involved 121 nurses (not part of the study sample). The data were collected and subjected to factor analysis as a useful and widely accepted technique to test construct validity. A panel consisting of six (6) experts was established comprising of two (2) experts from Sana’a University, two (2) from a health institute, and two (2) nurse practitioners from hospitals were to assess the content validity of the questionnaire. Feedback and comments from the expert panel regarding the instrument’s layout and format, relevance, accuracy, consistency, and scoring system were assessed with corrections carried out prior to use in the main study. A pre-test for comprehension [understandability] of the questions was also conducted among 20 nurses (not participating in the study). The instrument’s reliability was tested via Cronbach’s alpha since it is the most widely-used measure of internal consistency in assessing the questionnaire’s items. The result of this test was 0.81 for the knowledge section and 0.79 for the practice section, which is acceptable.

### Data collection

Data were collected from the three groups at the three stages [points of time] during the study period. Pre-evaluation data (T1) were collected before conducting the intervention and before distributing the educational materials. Post-intervention evaluation data (T2) were obtained immediately following the end of the intervention. Follow-up evaluation data (T3) were collected after 3 months upon completion of the intervention programme. The data were collected on the same day of distributing the questionnaires to avoid any exchange of information among the participants. A unique identification number was allocated to each questionnaire in order to track each participant without revealing their identity during the data collection and analysis phase. The dropout rate was 9% at T3; though this result is not high compared to other studies. The reasons may have been attributed to transferring to another city given the current conflict [war] situation in the country or the inability to reconcile his/her practical responsibilities and training time. Data were collected between 1st May and 30th October 2016.

### Data analysis

The Statistical Package for Social Sciences (SPSS), version 22.0 was used to enter, clean, check, and examine the collected data. A Chi-square test and Kruskal-Wallis test (non-parametric tests) were performed to test the difference between the groups at baseline.

The generalised estimating equation (GEE) was used to analyse the primary outcome variables, specifying each group as the between-subjects factor and time as the within-subjects factor, with the previous training course and previous experience covariates for all repeated-measures analysis. The Wald Chi-Square test was used to examine the time, group, and interaction (time*group) effect and to estimate the mean scores of the study variables over time. A simple main effects test was conducted whenever a significant interaction effect was found. A relatively straightforward post-hoc analysis (pairwise) was also conducted to examine differences between the group effect and the within group effect over time. The statistical significance was reported at a *P-*value of less than 0.05 level (two-tailed) with a 95% confidence interval.

## Results

### Participants’ demographic profile

In this study, Kruskal-Wallis and Ch-squire tests were conducted to assess homogeneity between the study groups. The Kruskal-Wallis test results showed no statistically significant difference in the Mean ranks between these groups related to the respondents’ age. Similarly, the results of the Chi-square test indicated that there was no statistically significant difference between the groups in the number and percentage regarding the respondents’ gender, previous in-service training courses, date of last training course attended, previous working experience and years of working experience. Accordingly, this means that the three groups were homogeneous in their demographic characteristics. The results for all the above tests are presented in Table 2.

**Table 2:** Differences between the study groups related to the participants’ socio-demographic data

Variables	Intervention1 (*n* = 180)		Intervention2 (*n =* 180)		Waitlist group (*n =* 180)		χ^**2**^	***P-value***
	**Mean ranks**		**Mean ranks**		**Mean ranks**			
**Age**	255.92		274.12		281.46		2.576	***0.28***
	**No.**	**%**	**No.**	**%**	**No.**	**%**		
**Gender**								
Male	87	48.3	100	55.6	93	51.7	1.88	**0.39**
Female	93	51.7	80	44.4	87	48.3		
**Previous in-service training**								
Yes	43	23.9	44	24.4	49	27.2	0.609	**0.74**
No	137	76.1	136	75.6	131	72.8		
**Date of last training course attended**								
≤ 1 year	25	55.6	25	56.8	21	44.7	1.644	**0.44**
> 1 year	20	44.4	19	43.2	26	55.3		
**Previous working experiences**								
Yes	139	77.2	135	75.0	130	72.2	1.199	**0.55**
No	41	22.8	45	25.0	50	27.8		
**Years of working experiences**								
< 5	80	44.4	76	42.2	66	36.7	2.387	**0.30**
≥ 5	100	55.6	104	57.8	114	63.3		

* χ2 is significant at ≤0.05 level

### Differences between the study groups

The homogeneity within the demographic variables and the study outcomes at the baseline between the three groups was next examined. The results revealed no statistically significant differences related to these aspects. Moreover, due to the moderately low response rate at time (3), further analysis was carried out to exclude any differences related to demographic characteristics and outcomes at the baseline between those nurses who completed the study and those who did not. The results also showed that there were no statistically significant differences.

### Effect on nurses’ knowledge of NI control measures

The analysis results showed a significant interaction effect regarding the knowledge score (χ2(4) = 281.05, *P* = 0.001). This result indicates that the group effect varied across time. The simple main effects analysis results also revealed that the estimated marginal mean for the knowledge scores of the intervention groups was sorted increasingly over time as anticipated. Furthermore, the between-subjects analysis results showed that the study groups did not differ in their mean knowledge scores at T1 (*P* = 1.00). The results of the simple effects post-hoc tests also revealed a statistically significant difference in the mean knowledge scores between the intervention groups at T2 (MD = 4.31, *P* < 0.001) and at T3 (MD = 4.48, *P* < 0.001) as compared to the waitlist group. This result indicates that participation in both intervention groups produced a knowledge gain detectable immediately post-intervention and at three (3) months following the end of the intervention.

However, the participants’ level of knowledge in both groups was significantly different in the three points of time. Here, the results of the within-subjects analysis showed that the waitlist group’s mean knowledge scores were not significantly different across the three points of time. As shown in Table 3, the results of the simple effects post-hoc analysis showed a statistically significant improvement in the mean knowledge score for the intervention groups from T1 to T2 and T1 to T3 (*P* < .001). Nevertheless, it is worth noting that the mean knowledge score for intervention-1 at T2 and T3 was significantly and substantially higher than that of intervention-2. From this, it can be inferred that intervention-1 was more effective in increasing the knowledge of NI control measures compared to intervention-2.

**Table 3:** Mean differences in nurses’ knowledge scores between intervention groups compared to waitlist group at baseline, six weeks and three months after intervention

Intervention Groups	Assessment time	knowledge Means		Mean Difference	Std. Error	df	***P-value***
		Intervention Groups	Wait-list Group				
**Intervention-1**	**Time-1**	14.96	15.36	0.40	0.374	1	**1.000**
	**Time-2**	23.68	17.15	6.52	0.346	1	< 0.001*
	**Time-3**	23.87	17.02	6.86	0.318	1	< 0.001*
**Intervention-2**	**Time-1**	15.18	15.36	0.17	0.340	1	**1.000**
	**Time-2**	19.37	17.15	2.21	0.307	1	< 0.001*
	**Time-3**	19.39	17.02	2.38	0.298	1	< 0.001*

- * *= P*-value is significant at ≤0.05 level

### Effect on nurses’ practices of NI control measures

Similarly, the results here showed a significant interaction effect on the practice score (χ2(4) = 180.52, *P* = 0.001). The results of the simple main effects analysis indicated that the estimated marginal mean for the practice scores for intervention group-1 and intervention group-2 increased from T1 (Mean = 7.63 and 8.03) to T2 (Mean = 11.62 and 8.88), respectively and also from T1 (Mean = 7.63 and 8.03) to T3 (Mean = 11.46 and 9.00), respectively. The between-subjects results showed that the two intervention groups did not differ in their mean knowledge scores at T1 (*P* = 0.990). Likewise, the waitlist group and the two intervention groups did not vary nor differ in their mean practice scores at T1 (*P* = 1.000).

However, the results of the simple effects post-hoc tests showed a statistically significant difference in the mean practice scores between the intervention groups at T2 (MD = 2.74, *P* < 0.001) compared to the waitlist group. The same tests’ results also showed that the mean difference in the practice scores between the intervention groups was statistically significant at T3 (MD = 2.46, *P <* 0.001) compared to the waitlist group. The within-subjects analysis showed no significant difference in the mean practice scores in the waitlist group over the three points of time. Although, the results of the simple effects post-hoc analysis showed a statistically significant increase in the mean practice score for intervention-1 from T1 to T2 and T1 to T3 (*P* < 0.001), although the mean practice score for intervention-2 did not increase significantly from T1 to T2 (*P* = 0.061); though it increased significantly from T1 to T3 (*P* = 0.020). Notably, this increase was not enough to reach a statistically significant level compared to the waitlist group. Accordingly, it can be inferred that intervention-1 was more effective in increasing the practice of NI control measures than intervention-2. Further details are provided in Table 4.

**Table 4:** Mean differences in nurses’ practice scores between intervention groups compared to waitlist group at baseline, six weeks and three months after intervention

Intervention Groups	Assessment time	Practice Means		Mean Difference	Std. Error	df	***P-value***
		Intervention Groups	Wait-list Group				
**Intervention-1**	**Time-1**	7.63	7.96	0.33	0.270	1	**1.000**
	**Time-2**	11.62	8.00	3.62	0.204	1	< 0.001*
	**Time-3**	11.46	8.01	3.45	0.253	1	< 0.001*
**Intervention-2**	**Time-1**	8.08	7.96	0.07	0.241	1	**1.000**
	**Time-2**	8.88	8.00	0.88	0.280	1	**0.061**
	**Time-3**	9.00	8.01	0.99	0.287	1	0.020*

- * *= P*-value is significant at ≤0.05 level

## Discussion

The results of the study provided evidence that participation in the intervention groups produced a significant improvement in the mean knowledge scores of NI control measures over the three points of time compared to the waitlist group. However, the mean knowledge score for intervention group-1 was significantly higher than intervention group-2. The difference in the mean knowledge score between both intervention groups was significant at T2 (MD = 4.31, *P* < 0.001) and T3 (MD = 4.48, *P* < 0.001) compared to the waitlist group. This indicates that intervention-1 was more effective in increasing nurses’ knowledge of NI control measures than intervention-2 at T2 and T3. The effectiveness of intervention-1, in this case, could be attributed to the interactive educational sessions that were received by the participants. It could also be attributed to the improvement in their understanding of the content and ability to discover knowledge on their own compared to those who received only the educational module “without training” in intervention group-2.

It was also worth noting that the gain in knowledge achieved by both intervention groups was sustained over the 3 months following the end of the intervention. However, the participants’ knowledge in intervention group-1 at T3 was higher compared to the participants in intervention group-2. This further implies that intervention-1 (“face-to-face training course” + module) was better in improving nurses’ knowledge of NI control measures compared to intervention-2 (module only “without training”).

Our findings are consistent with previous results of one group pre-test and post-test studies aiming to evaluate the effectiveness of the educational intervention in infection control measures to improve the level of knowledge among nursing students and nurses [17, 26–29]. These studies showed a significant improvement in the mean score attributed to knowledge in the post-intervention compared to the pre-intervention evaluation.

Likewise, this study’s results are consistent with the results of other studies using experimental and control groups to evaluate the effectiveness of an infection control educational intervention to improve the knowledge of nurses [19, 30, 31]. Such studies also reported a significant improvement in nurses’ knowledge regarding infection control measures among the intervention groups compared to the control groups. A possible explanation for the improvement in nurses’ knowledge could be that the education programme or process was effective in informing and convincing the nurses that infection control measures were important, thereby improving their knowledge.

Concerning the practice of nurses, the results of the present study showed that intervention-1 had a significant positive effect on the practices of nurses regarding NI control measures immediately post-intervention compared to the waitlist group. In contrast, although the mean practice score for intervention group-2 increased from T1 to T2, this increase was not sufficient to reach a statistically significant level compared to the waitlist group. Further, it was noted that immediately post-intervention, the mean practice score for intervention group-1 was significantly higher than in intervention group-2. This implies that intervention group-1 was more effective in increasing the practice of nurses regarding NI control measures than in intervention group-2, which was detectable immediately following the intervention.

Here, the effectiveness of intervention group-1 could be attributed to the face-to-face training course and the hands-on aspects of the course that were received compared to nurses that did not receive the training in intervention group-2. These results corroborate with the findings of many reviewed studies [17–19, 26–30, 32]. Such studies identified an immediate improvement among those participants who had received training compared to those who did not receive any form of training after an educational intervention.

On the other hand, although the improvement in the practice scores from T1 to T3 for both intervention-1 and intervention-2 was shown to be significant (*P* < 0.001 and *P* < 0.020), respectively, the mean practice score for intervention-1 was higher than in intervention-2. Accordingly, this provides further support that intervention group-1 was more effective in increasing the nurses’ practices of NI control measures immediately post-intervention and 3 months following the end of the intervention compared to intervention group-2. This result agrees with the findings of several similar studies [19, 30, 33] revealing an evident improvement over the three points of time of evaluation.

There were several limitations inherent in the current study that should be addressed in future studies. One limitation concerns the scope of the study, which was exclusive to public hospitals, with nurses who held a three-year nursing diploma and was undertaken in one region in the north of the Republic of Yemen. Therefore, the generalisability of the findings should be considered cautiously. Accordingly, further studies involving different hospitals, all nurse categories and other settings in Yemen are recommended. Also recommended in further research is employing direct observation to assess the practice of nurses regarding NI control measures. Moreover, intervention group-2 and the waitlist group only received and had access to the educational materials and did not receive the training course at the conclusion of the study.

## Conclusion

Based on the findings of this study, it can be concluded that intervention-1 (face-to-face training + module) and intervention-2 (module only) had significant positive effects the knowledge and practices of nurses’ regarding NI control measures over the three points of time. However, intervention-1 effectively improved nurses’ knowledge and enhanced their skills in applying NI control measures in dealing with NI control issues. Therefore, it can be concluded that intervention-1 can be used for nurses in the form of an in-service training course conducted on a regular basis, at least six monthly. Furthermore, it could also be incorporated in nursing curricula of high institutes of health sciences in Yemen.

## Supplementary Information

**Additional file 1: Table A.** The number of nurses selected from each hospital

## Data Availability

It was stated in the respondents’ consent form that data used in the current study would remain confidential and would not be shared publicly. However, data are available from the corresponding author on reasonable request.

## Abbreviations

NIs: Nosocomial Infections
ICC: Intra-Cluster Coefficient;
GEE: Generalised Estimating Equation
SPSS: Statistical Package for Social Sciences
WHO: World Health Organisation
CSSD: Central Sterile Supply Department
MD: Mean Difference
HCW: Healthcare workers
ICP: Infection control practitioner.
