# Supplementary material for: Implementation of an educational module on nosocomial infection control measures: a randomised hospital-based trial
Source: BMC Nurs. 2021 Feb 17;20:33. doi: 10.1186/s12912-021-00551-0 (PMC7890621; doi:10.1186/s12912-021-00551-0)
Supplement: Supplementary file 1 — Additional file 1: Table A. The number of nurses selected from each hospital [file 12912_2021_551_MOESM1_ESM.docx]

**Table A: The number of nurses selected from each hospital**

| Status | Hospital’s name | Hospital’s nurses | Required sample | | Total in each arm |
| --- | --- | --- | --- | --- | --- |
|  |  |  | (%) | (no.) |  |
| Intervention1 | Al-Thwara H | 602 | 86 | 154 | 180 |
|  | Al-Kuwait H | 100 | 14 | 26 |  |
| Intervention2 | Al-Jomhory H | 150 | 55 | 100 | 180 |
|  | Dhamar H | 121 | 45 | 80 |  |
| Waitlist group | Dhul’a H | 40 | 14 | 25 | 180 |
|  | Al-Sab'een H | 119 | 41 | 74 |  |
|  | 26 September H | 60 | 21 | 37 |  |
|  | Al-wahda H | 70 | 24 | 44 |  |
| Total number | 8 | 1262 | 100 | 540 | 540 |

* H= Hospital
